# Association between Smoking Behavior Patterns and Glycated Hemoglobin Levels in a General Population

**DOI:** 10.3390/ijerph15102260

**Published:** 2018-10-16

**Authors:** Dong-Woo Choi, Jooeun Jeon, Sang Ah Lee, Kyu-Tae Han, Eun-Cheol Park, Sung-In Jang

**Affiliations:** 1 Department of Public Health, Graduate School, Yonsei University, Seoul 03722, Korea; cdw6027@yuhs.ac (D.-W.C.); jjooeun@yuhs.ac (J.J.); ivory0817@yuhs.ac (S.A.L.); 2 Institute of Health Services Research, Yonsei University, Seoul 03722, Korea; ecpark@yuhs.ac; 3 Department of Preventive Medicine, Yonsei University College of Medicine, Seoul 03722, Korea; kthan.phd@gmail.com; 4 Department of Policy Research Affairs, National Health Insurance Service Ilsan Hospital, Koyang 10444, Korea

**Keywords:** HbA1c, dual smoking, type 2 diabetes mellitus, KNHANES

## Abstract

This study investigated the association of smoking behaviors, including dual smoking (smoking both cigarettes and e-cigarettes), cigarettes smoking, and previous smoking, with glycated hemoglobin (HbA1c) levels. National Health and Nutrition Examination Survey (KNHANES) data from 2014–2016 was used. Associations between smoking behavior patterns and HbA1c levels were analyzed via multiple regression. Among 8809 participants, individuals who were dual smokers and cigarettes smokers had significantly higher HbA1c levels than non-smokers (dual: β = 0.1116, *p* = 0.0012, single: β = 0.0752, *p* = 0.0022). This relationship strengthened in subgroups of men (dual: β = 0.1290, *p* = 0.0013, single: β = 0.1020, *p* = 0.0014, ex: β = 0.0654, *p* = 0.0308), physically inactive subjects (dual: β = 0.1527, *p* = 0.0053, single: β = 0.0876, *p* = 0.0197), and overweight (dual: β = 0.1425, *p* = 0.0133) and obese individuals (dual: β = 0.1694, *p* = 0.0061, single: β = 0.1035, *p* = 0.0217). This study suggests that smoking behaviors are likely to increase the risk of HbA1c level in a general population. The health effects of dual smoking remain uncertain and should be addressed in the future.

## 1. Introduction

Glycated hemoglobin (HbA1c) is used as one of the standard measures to diagnose diabetes worldwide. HbA1c has several advantages as a diagnostic method compared with a fasting glucose test, and has been recommended for the determination of glucose control among people who have already diagnosed with diabetes [1,2,3]. The HbA1c level represents the two to three month average blood glucose level and is therefore used to monitor glycemic control in patients with diabetes [4,5].

Cigarette smoking has been identified as a risk factor for mortality and as the second leading risk factor for early death and disability worldwide [6]. Additionally, individuals with diabetes who smoke are more likely to experience difficulty with insulin dosing and disease control than non-smokers [6]. Notably, previous studies revealed that when compared to non-smokers, cigarette smokers had elevated levels of HbA1c, as well as a 30–40% higher risk of type 2 diabetes [7,8,9]. Moreover, the prevalence of e-cigarette use in South Korea was 7.1% for men and 1.2% for women in 2015, which had increased by 2.7% in men and 0.8% in women compared to 2014 [10]. Electronic cigarettes (e-cigarettes), which aerosolize nicotine to produce a vapor that purportedly contains fewer traditional toxins than secondhand smoke, are used to emulate cigarette smoking. These devices are popular for smoking cessation and reduction among those who wish to eliminate or reduce cigarette use [11,12,13], and several studies have reported the positive effects of e-cigarettes for this purpose [14,15]. However, the effects of e-cigarettes use have not been as fully elucidated as those of cigarettes, and it remains uncertain whether these devices are safe for use by individuals interested in smoking cessation and reduction. Moreover, dual smoking, which includes using both e-cigarettes and ordinary cigarettes, has been revealed to likely induce tobacco dependence, although its other health effects are still unknown [16].

Unlike evidence related to cigarette smoking, there is insufficient evidence to clarify the relationship between dual smoking or e-cigarettes smoking and HbA1c levels. Therefore, this study investigated the association of various smoking behaviors, including dual smoking, cigarettes alone, and previous smoking, with HbA1c levels in a general population.

## 2. Methods

### 2.1. Study Population

This cross-sectional study was based on data from the 2014–2016 Korea National Health and Nutrition Examination Survey (KNHANES), a nationwide survey conducted by the Korea Centers for Disease Control (KCDC) and Prevention. From a database of 23,080 participants, we excluded patients who had received a diagnosis of diabetes from a physician, had used medication for diabetes, and had HbA1c levels < 6.5% (48 mmol/mol). We also excluded participants for whom information was unavailable regarding smoking behavior, HbA1c level, age, sex, occupation, household income, educational level, physical activity, body mass index (BMI), alcoholic behavior, pack-years of smoking, anemia status, family history of diabetes mellitus, and caloric intake. Finally, a total of 8,809 participants were included in this study.

### 2.2. Variables

The percent HbA1c was set as the dependent variable and was determined using high performance liquid chromatography. Smoking behavior, which combined cigarette and e-cigarette use, was the primary independent variable. The survey asked all subjects whether they used cigarettes or e-cigarettes currently or had ever used these products during their lifetime. Accordingly, we classified the subjects into four categories: dual smokers (both cigarettes and e-cigarette), single smokers (cigarettes smokers), former smokers (ex-smokers), and non-smokers.

The covariates included age, sex, occupation, household income, educational level, BMI, physical activity, pack-years of cigarette smoking, alcoholic behavior, family history of diabetes mellitus, year, and caloric intake. Age was stratified into 10-year periods of 20–29, 30–39, 40–49, 50–59, 60–69, and ≥70 years. Socioeconomic status was stratified into four groups by household income level from 1 quartile (low) to 4 quartiles (high). Obesity was defined as a BMI ≥ 25. Physical activity, which was self-reported, was stratified by the World Health Organization recommendations (WHO) that adults should perform at least 150 min of moderate-intensity or 75 min of vigorous-intensity aerobic physical activity, or an equivalent combination of both types, throughout the week. Pack year of cigarette smoking for the lifetime was calculated by multiplying the daily smoking amount by the smoking period by referring to the previous study. Pack-year of cigarette smoking for the lifetime was calculated by multiplying the daily smoking amount by the smoking period by referring to the previous study which classified this data as none, light (≤26.7 pack-years), medium (26.8–40.4 pack-years), heavy (40.5–55.5 pack-years), and very heavy (>55.5 pack-years) [17,18]. Alcoholic behavior was defined as the intake of such beverages above 1 times/day, 1–4 times per month, and ≥2 times/week. A family history of diabetes was defined as having an immediate family member (e.g., father, mother, brother, and/or sister) with type 1 or type 2 diabetes. The caloric intake was determined as the number of kcals consumed per day, which was calculated by multiplying 4 kcal/g by intake of carbohydrates and protein and 9 kcal/g by intake of fat, divided by the total energy consumed during the day, and then multiplied by 100.

### 2.3. Statistical Analysis

For all analyses, we used the sampling weights variable provided by KNHANES. First, we examined the distribution of the study population in terms of the frequency and percentage of each categorized variable, and then determined the mean and standard deviation for the distribution of each continuous variable. The Kolmogorov-Smirnov goodness-of-fit test was used to test the normality of distribution for HbA1c. As this variable was not normally distributed, we excluded outliers to improve normality. Next, we used an analysis of variance (ANOVA) to compare the average HbA1c levels according to independent variables. A multiple regression analysis including weight as a variable was used to estimate the association between smoking behavior patterns and HbA1c after controlling for age, sex, occupation, household income, educational level, physical activity, BMI, alcoholic behavior, pack-years of smoking, family history of diabetes mellitus, year, and caloric intake. Finally, we performed the multiple regression analysis of subgroups stratified by sex and physical activity, and BMI. All statistical analyses were performed using SAS version 9.4 (SAS Institute, Inc., Cary, NC, USA). A *p*-value < 0.05 was considered statistically significant.

### 2.4. Ethical Statement

The Korea National Health and Nutrition Examination Survey data are openly published, thus, ethical approval was not required for this study. This study did not require informed consent from the participants, as their information was fully anonymized and unidentified prior to analysis.

## 3. Results

Table 1 presents the distribution of the study population. Regarding smoking behaviors, dual smokers, single smokers, ex-smokers, and non-smokers accounted for 1.61% (*n* = 142), 15.43% (*n* = 1359), 18.78% (*n* = 1654), and 64.18% (*n* = 5654) of the sample, respectively. Men and women comprised 39.99% (*n* = 3523) and 60.01% (*n* = 5286) of the sample, respectively. Normal weight/underweight, overweight, and obese subjects comprised 46.48% (*n* = 4094), 22.90% (*n* = 2017), and 30.63% (*n* = 2698) of the sample, respectively. The overall mean HbA1c level was 5.48 ± 0.27%.

Table 2 demonstrates the associations of smoking behavior with covariates and HbA1c levels. Of the smoking behavior groups, dual smokers and single smokers had significantly higher HbA1c levels than non-smokers (dual: β = 0.1116, *p* = 0.0012, single: β = 0.0752, *p* = 0.0022). HbA1c levels tended to increase significantly with age (30–39 years: β = 0.0856, 40–49: β = 0.1538, 50–59: β = 0.2648, 60–69: β = 0.3477, and ≥70: β = 0.4147, *p* < 0.0001 vs. 20–29 years), BMI (overweight: β = 0.0547 and obesity: β = 0.1457, *p* < 0.0001 vs. normal/underweight), education level (high school: β = 0.0218, *p* = 0.0361, university or college: β = 0.0217, *p* = 0.0221 vs. graduated school), and physical activity (no: β = 0.0121, *p* = 0.0079 vs. yes). However, household income, occupation, pack-years of cigarette smoking, and caloric intake had no significant effect on this association.

**Figure 1:**
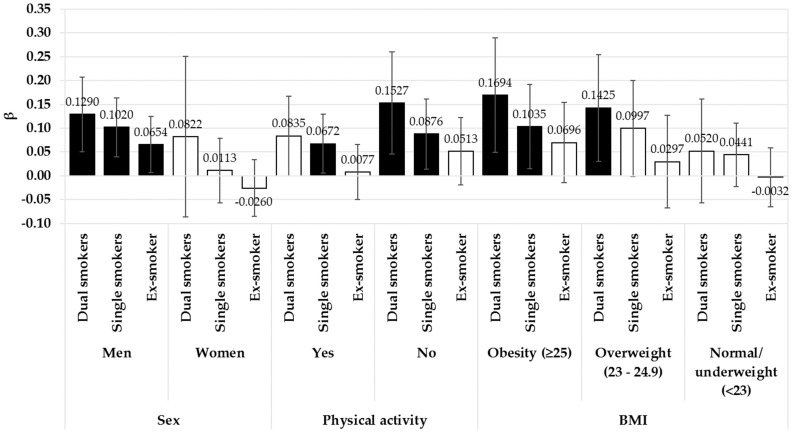
The results of subgroup analysis with multiple regression to investigate the association between smoking behavior and HbA1c according to sex, physical activity, and BMI. Black bars are statistically significant results (*p* < 0.05). Non-smokers were the reference group. Analyses were adjusted for the following covariates: age, sex, occupation, household income, educational level, BMI, physical activity, pack-years of cigarette smoking, alcoholic behavior, family history of diabetes mellitus, year, and caloric intake.

Figure 1 presents the results of subgroup analyses according to sex, physical activity, and BMI. Men who were dual, single, and ex-smokers had significantly higher HbA1c levels, compared to non-smokers (dual: β = 0.1290, *p* = 0.0013, single: β = 0.1020, *p* = 0.0014, ex-smoker: β = 0.654, *p* = 0.0308). Among females, there was no difference in HbA1c levels across the exposure categories. Physically inactive subjects who were dual and single smokers had significantly higher HbA1c levels than non-smokers (dual: β = 0.1527, *p* = 0.0053, single: β = 0.0876, *p* = 0.0197). Subjects who were single smokers and performed physical activity according to WHO recommendations also had significantly higher HbA1c levels than non-smokers (β = 0.0672, *p* = 0.0344). Among obese participants, dual and single smokers had significantly higher HbA1c levels, compared to non-smokers (dual: β = 0.1694, *p* = 0.0061, single: β = 0.1035, *p* = 0.0217). Subjects who were dual smokers and overweight exhibited higher HbA1c levels than non-smokers (β = 0.1425, *p* = 0.0133). However, there were no significant results in normal/underweight subjects.

## 4. Discussion

In this study of a general population, we observed elevated HbA1c levels among subjects who were both dual and single smokers compared to among non-smokers. These relationships were particularly strong among male, physically inactive, and obese subjects.

Cigarette smoking has been revealed to increase HbA1c levels in patients without diabetes. One of the previous studies revealed that cigarette smoking was associated with an increase in HbA1c in the general population [19,20]. In addition, one study found that e-cigarettes smokers are likely to have a high HbA1c level compared to cigarette smokers [21]. A possible explanation for this could be the increase in the rate of glycation of HbA1c induced by exposure to glycotoxin from cigarette smoke or the relatively high degree of tissue hypoxia [22]. Therefore, these results support our findings regarding the effects of dual cigarette smoking in our sample. However, we were unable to determine direct associations between e-cigarette smoking and HbA1c levels.

In the analyses stratified by sex, men who were dual and single smokers had higher HbA1c levels than non-smokers, whereas among women, there were no significant results. Although no clear evidence supports a link between dual smoking and increased HbA1c levels, several studies have supported the association between cigarette smoking and HbA1c levels. One of the previous studies revealed that cigarette smoking was independently associated with higher HbA1c concentrations in both men and women [8]. Evidence suggests a dose-response relationship with the number of cigarettes smoked among current smokers, and an inverse association of the number of years since smoking cessation with HbA1c levels has been observed among male ex-smokers.

In our analysis, we also stratified smoking behaviors by physical activity levels. Among physically inactive subjects, dual smokers who participated in both cigarette smoking and e-cigarette smoking were more strongly associated with HbA1c levels. Potentially, lifestyle factors such as physical inactivity and dual smoking act synergistically to elevate HbA1c levels. The ability of physical activity to reduce HbA1c levels is well-known, and regular physical activity is recommended by healthcare providers or professionals as a beneficial practice [23,24,25]. These results may corroborate our findings of higher HbA1c levels among dual smokers who were smoking both e-cigarettes and cigarettes than among non-smokers among physically active subjects. However, it remains unclear whether e-cigarette smoking alone can induce an increase in more HbA1c levels. Future studies should investigate the effects of e-cigarette use on HbA1c levels.

According to BMI, dual smokers had a strong association of elevated HbA1c levels among people who were obese and overweight compared to those who were normal and underweight. These results may be due to the fact that smoking is likely to increase obesity-related comorbidities associated with visceral adiposity. Therefore, smokers tended to be obese compared to non-smokers [26]. Moreover, a previous study revealed that visceral adiposity was associated with HbA1c [27]. We guess that these potential biological mechanisms are underlying the strong association between dual smoking and elevated HbA1c levels compared to non-smoking among obese people compared to normal and underweight people.

This study had several limitations of note. First, as this was a cross-sectional study, the causal relationship between smoking behavior patterns and HbA1c levels should be interpreted cautiously. Second, we were not able to consider the type of e-cigarette, frequency of vaping, or concentration of nicotine. Third, data regarding smoking behavior, health behavior, and socioeconomic status were collected via self-report surveys and thus might have been subject to recall bias and underestimated smoking behaviors. Fourth, caloric intake was measured indirectly. Fifth, we could not find references about association between dual smoking and HbA1c although we tried to find previous studies. Therefore, we could not provide enough discussion about dual smoking. Sixth, we could not consider each single e-cigarette smoking behavior because the number of single e-cigarette smokers was very small. Therefore, further study should consider each single smoking behavior. Finally, we may not have fully accounted for confounding factors in our analysis.

Despite these limitations, however, our study had several strengths. First, our analysis used HbA1c levels which reflect average plasma glucose over the previous eight to twelve weeks and are able to be measured at any time of day, regardless of the duration of fasting. Second, HbA1c levels were measured using clinical tests, therefore producing reliable and clear data. Third, few other studies have evaluated the association between smoking behavior, including e-cigarettes, and HbA1c levels. Thus, this study might provide the impetus to seek the association between smoking behavior, especially dual smoking, and HbA1c. Finally, our study adjusted several social factors known as potential confounders for smoking behaviors or HbA1c, including sex, socioeconomic status, and health behaviors, to appropriately estimate associations among smoking behavior patterns.

## 5. Conclusions

Smoking behaviors, especially dual smokers who use e-cigarettes and cigarettes, were found to likely result in increased HbA1c levels in a general population, particularly among male, physically inactive, and obese adults. This study is one of the first to attempt to examine the combined and individual effects of cigarettes and e-cigarettes on HbA1c levels.

We note that unlike cigarettes, the health effects of dual smoking have not been fully elucidated. Although we found that dual smoking both cigarettes and e-cigarettes is unhealthy, it remains unclear whether e-cigarette use affects other health outcomes. Therefore, more studies should explore the adverse health effects of e-cigarettes specifically and/or that we should educate physicians and their patients about the risks of e-cigarettes. This may be especially important since many people believe that e-cigarettes are “safe”.

## Figures and Tables

**Table 1:** The general characteristics of study population.

Variables	N/Mean	%/SD	HbA1c		
			Mean	SD	*p*-Value
**Smoking Behavior**					<0.0001
Dual smokers	142	1.61	5.48	±0.34	
Single smokers	1359	15.43	5.50	±0.27	
Ex-smoker	1654	18.78	5.51	±0.27	
Non-smoker	5654	64.18	5.47	±0.27	
**Sex**					0.7098
Men	3523	39.99	5.50	±0.27	
Women	5286	60.01	5.48	±0.27	
**Age (years)**					<0.0001
20–30	1176	13.35	5.26	±0.21	
30–39	1930	21.91	5.37	±0.23	
40–49	1772	20.12	5.44	±0.23	
50–59	1692	19.21	5.57	±0.23	
60–69	1314	14.92	5.65	±0.24	
≥70	925	10.50	5.70	±0.24	
**Household Income**					0.057
Low	2821	32.02	5.45	±0.26	
Mid-low	2641	29.98	5.46	±0.26	
Mid-high	2122	24.09	5.50	±0.27	
High	1225	13.91	5.59	±0.28	
**Occupational Categories**					0.0913
White collar worker	2371	26.92	5.41	±0.25	
Pink collar worker	1154	13.10	5.47	±0.26	
Blue collar worker	1901	21.58	5.55	±0.26	
Unemployed	3383	38.40	5.50	±0.28	
**Educational Level**					0.0703
High school	4455	50.57	5.56	±0.26	
University or College	3831	43.49	5.40	±0.25	
Graduated school	523	5.94	5.42	±0.26	
**BMI**					<0.0001
Normal/underweight (<23)	4094	46.48	5.41	±0.25	
Overweight (23–24.9)	2017	22.90	5.50	±0.25	
Obesity (≥25)	2698	30.63	5.59	±0.27	
**Physical Activity**					0.1061
Yes	4495	51.03	5.46	±0.27	
No	4314	48.97	5.51	±0.27	
**Pack Year of Cigarette Smoking**					<0.0001
Very heavy	173	1.96	5.66	±0.28	
Heavy	188	2.13	5.65	±0.26	
Medium	474	5.38	5.63	±0.26	
Light	2248	25.52	5.46	±0.26	
None	5726	65.00	5.47	±0.27	
**Alcoholic Behavior**					<0.0001
less than 1 time for a month	3974	45.11	5.53	±0.27	
1–4 times for a month	3008	34.15	5.44	±0.26	
2 times or above for a week	1827	20.74	5.46	±0.27	
**Family History of Diabetes**					<0.0001
Present	1986	22.55	5.52	±0.27	
Absent	6823	77.45	5.47	±0.27	
**Year**					<0.0001
2014	2684	30.47	5.53	±0.27	
2015	2870	32.58	5.47	±0.27	
2016	3255	36.95	5.45	±0.27	
**Caloric Intake †**	2054.72	±940.62			
**Total**	8809	100.00	5.48	±0.27	

† Mean and Standard deviation (SD) of the continuous independent variables in this study.

**Table 2:** The results of multiple regression analysis to investigate the association between smoking behavior patterns and HbA1c.

Variables	HbA1c		
	β	SE	*p*-Value
**Smoking Behavior**			
Dual smokers	0.1116	0.0343	0.0012
Single smokers	0.0752	0.0245	0.0022
Ex-smoker	0.0261	0.0234	0.2647
Non-smoker	Ref	-	-
**Sex**			
Men	−0.0184	0.0072	0.0114
Women	Ref	-	-
**Age (years)**			
20–30	Ref	-	-
30–39	0.0856	0.0079	<0.0001
40–49	0.1538	0.0082	<0.0001
50–59	0.2648	0.0091	<0.0001
60–69	0.3477	0.0100	<0.0001
≥70	0.4147	0.0122	<0.0001
**Household Income**			
Low	Ref	-	-
Mid-low	0.0062	0.0088	0.4848
Mid-high	0.0090	0.0089	0.3126
High	0.0093	0.0091	0.3096
**Occupational Categories**			
White collar worker	−0.0019	0.0061	0.7514
Pink collar worker	0.0086	0.0074	0.2426
Blue collar worker	0.0089	0.0069	0.1998
Unemployed	Ref	-	-
**Educational Level**			
High school	0.0218	0.0104	0.0361
University or College	0.0217	0.0095	0.0221
Graduated school	Ref	-	-
**BMI**			
Normal or underweight (<23)	Ref	-	-
Overweight (23–24.9)	0.0547	0.0058	<0.0001
Obesity (≥25)	0.1457	0.0055	<0.0001
**Physical Activity**			
Yes	Ref	-	-
No	0.0121	0.0045	0.0079
**Pack Year of Cigarette Smoking**			
Very heavy	−0.0061	0.0315	0.8478
Heavy	0.0223	0.0294	0.4471
Medium	0.0447	0.0260	0.0861
Light	−0.0163	0.0235	0.4886
None	Ref	-	-
**Alcoholic Behavior**			
less than 1 time for a month	Ref	-	-
1–4 times for a month	−0.0282	0.0056	<0.0001
2 times or above for a week	−0.0628	0.0069	<0.0001
**Family History of Diabetes**			
Present	0.0530	0.0056	<0.0001
Absent	Ref	-	-
**Year**			
2014	0.0822	0.0062	<0.0001
2015	0.0078	0.0063	0.2141
2016	Ref	-	-
**Caloric intake**	0.0000	0.0000	0.4240
